# Hysteroscopic Treatment of Primary Endometrial Marginal Zone B-cell Lymphoma (MALToma)

**Published:** 2020-01-24

**Authors:** F Scrimin, G Magoga, G Di Lorenzo, F Romano, MC De Angelis, A Vitagliano, J Crugno, A Di Spiezio Sardo

**Affiliations:** Institute for Maternal and Child Health, IRCCS “Burlo Garofolo”, Trieste, Italy;; Istituto di Ginecologia e Obstetricia, Università Cattolica del Sacro Cuore, Rome, Italy;; Department of Public Health, School of Medicine, University of Naples Federico II, Naples, Italy;; Department of Women and Children’s Health, Unit of Gynecology and Obstetrics, University of Padua, Padua, 35128, Italy;; Obstetrics, Gynecology and Reproductive Sciences Department. University of Miami. Miller School of Medicine. Miami, FL USA.

**Keywords:** hysteroscopy, MALT, primary extranodal marginal zone B-cell lymphoma, polyp

## Abstract

Primary extranodal marginal zone B-cell lymphomas (MALToma) of the endometrium are rare tumors. We report a case of MALToma diagnosed within an endometrial polyp in a patient presenting with postmenopausal vaginal bleeding. The patient underwent a conventional hysteroscopic procedure for intrauterine polyps. There was no suspicion of malignancy during the preoperative investigations or at surgery. Conventional bipolar resection of the polyps was performed. The present case demonstrates that conservative management of endometrial MALToma may be considered as a safe alternative to hysterectomy. Further data on long-term follow up is needed to confirm the safety of this conservative approach.

## Introduction

Extranodal lymphoma refers to a group of neoplastic disorders arising from sites which normally do not contain : mucosal-associated lymphoid tissue (MALT). It can develop anywhere in the body, the skin, stomach, brain, small intestine and the Waldeyer’s ring being the most common sites (Zucca, 2008).

In this report, we discuss an exceptionally rare case of MALT lymphoma (MALToma) in a woman who presented with postmenopausal bleeding.

## Case report

A 60-year-old postmenopausal woman was referred to our Institution (Institute for Maternal and Child Health - IRCCS “Burlo Garofolo”, Trieste, Italy) complaining of postmenopausal vaginal bleeding. Her BMI was 28. She had a history of hypertension and smoke approximately five cigarettes a day. Her obstetrical history consisted of two vaginal deliveries, a first trimester spontaneous miscarriage and an ectopic pregnancy treated with salpingectomy.

**Figure 1:**
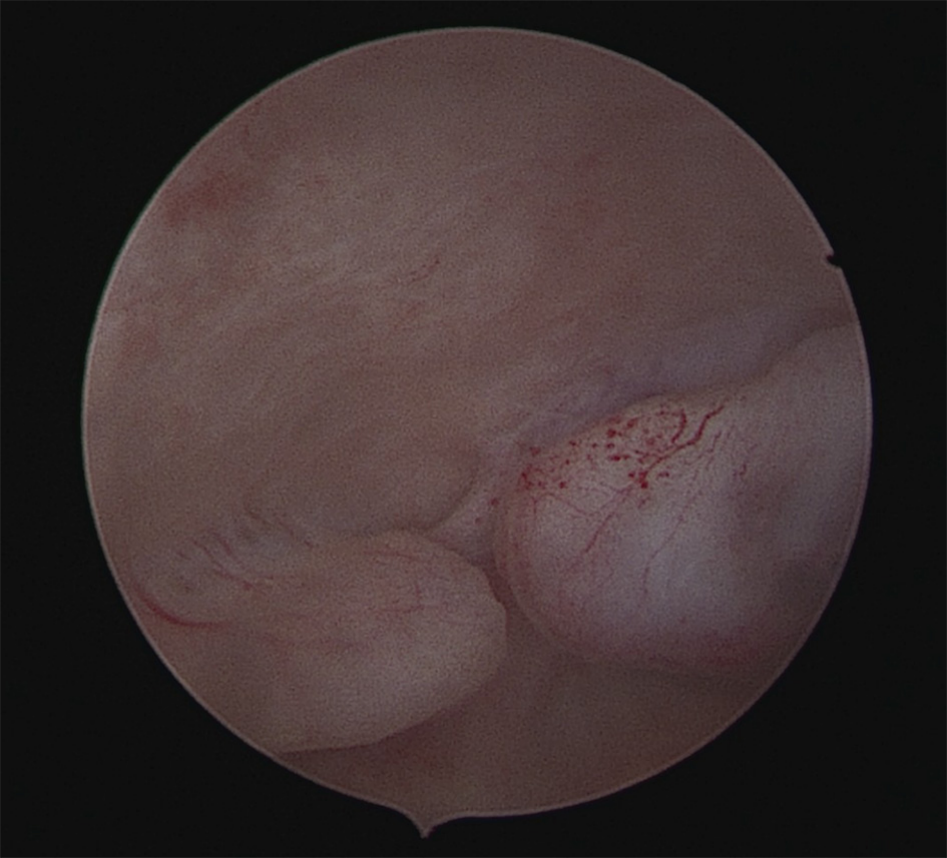
— Hysteroscopic view of the two endometrial polypoid lesions. Size are 6x4 mm and 10x5 mm; appearance is normal with typical vascularization .

The initial workup included a transvaginal ultrasound (TVS) that revealed a normal size uterus and bilateral normal adnexae; however, the endometrium appeared thickened with two focal hyperechoic, avascular areas of about 1 cm each, suggestive of endometrial polyps. The patient underwent an outpatient diagnostic hysteroscopy without anesthesia, using a 4 mm continuous-flow hysteroscope (Bettocchi Office Hysteroscope ‘size 4’, Karl Storz, Tuttlingen, Germany). However, the procedure was aborted due to intolerable pain. Following this, the patient was taken to the operating room for hysteroscopic polypectomy under anaesthesia. The procedure was performed using a 10 mm GYNECARE ® resectoscope with bipolar GYNECARE VERSAPOINT TM (Ethicon Inc., Somerville, NJ); saline solution was used at 50 - 70 mmHg automated constant intrauterine pressure regulated by Endomat (STORZ, Tuttlingen, Germany). The cervical canal was free of lesions, whereas in the uterine cavity two small polypoid lesions with a mean diameter of <1cm were identified. The remaining endometrium appeared atrophic. Both polypoid lesions were removed using bipolar electrical resection; the base of the lesion was sent to pathology as a separate specimen for analysis. Several random endometrial biopsies were also obtained (Figure 1).

**Figure 2:**
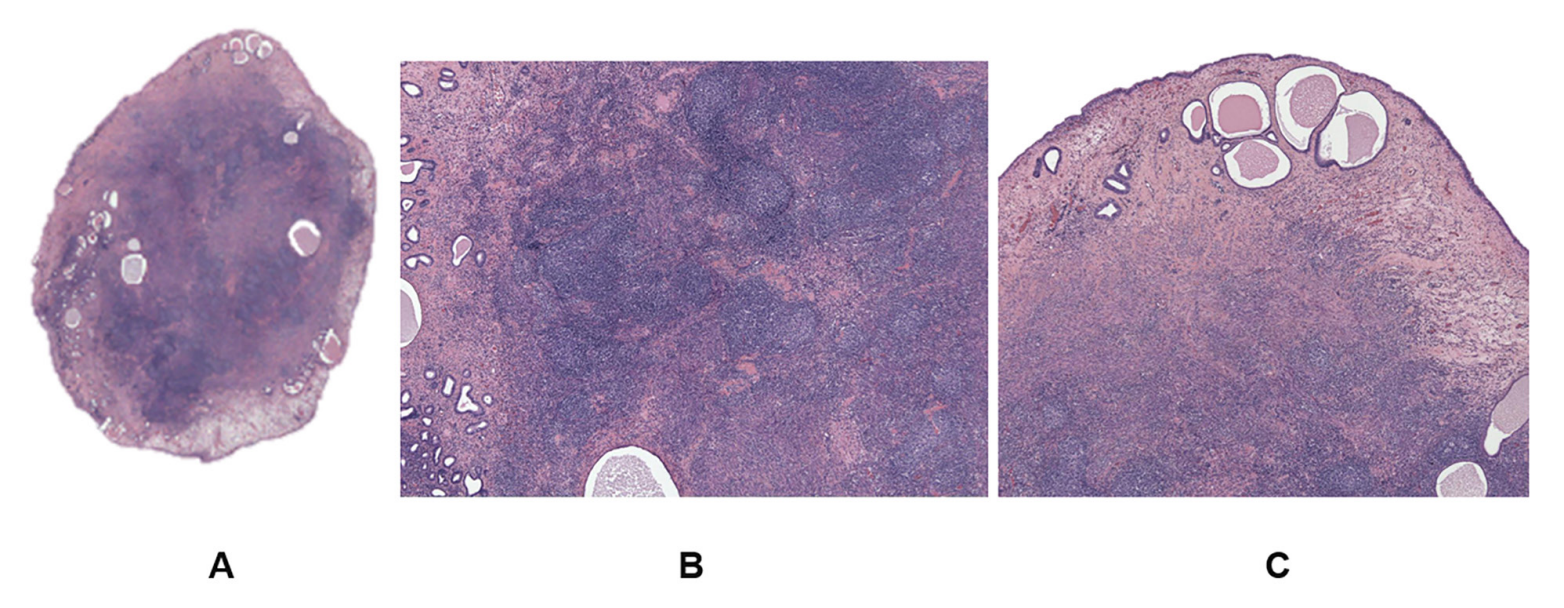
A) The endometrial polyp. Note the large number of cells in the middle of the lesion. Glands are located mainly in the periphery with areas peripheral edema. B) Germinal centers. C) MALT lymphoma inside the polyp, surrounded by normal epithelium.

Macroscopic examination showed two small endometrial polyps (6 x 4 and 10 x 5 mm in size) with a normal vascular pattern. Microscopic examination revealed two glandular endometrial polyps with the larger one containing lymphoid tissue consistent with extra-nodal marginal zone B-cell lymphoma of mucosa-associated lymphoid tissue (MALT lymphoma) (Figure 2 A and B). MALToma was located inside the stroma of the polyp surrounded by normal epithelium (Figure 2 C) with neoplastic cells confined to the polyp core, without invading the base of the lesion nor the surrounding endometrium. Endometrial biopsies were also negative for malignancy.

**Figure 3:**
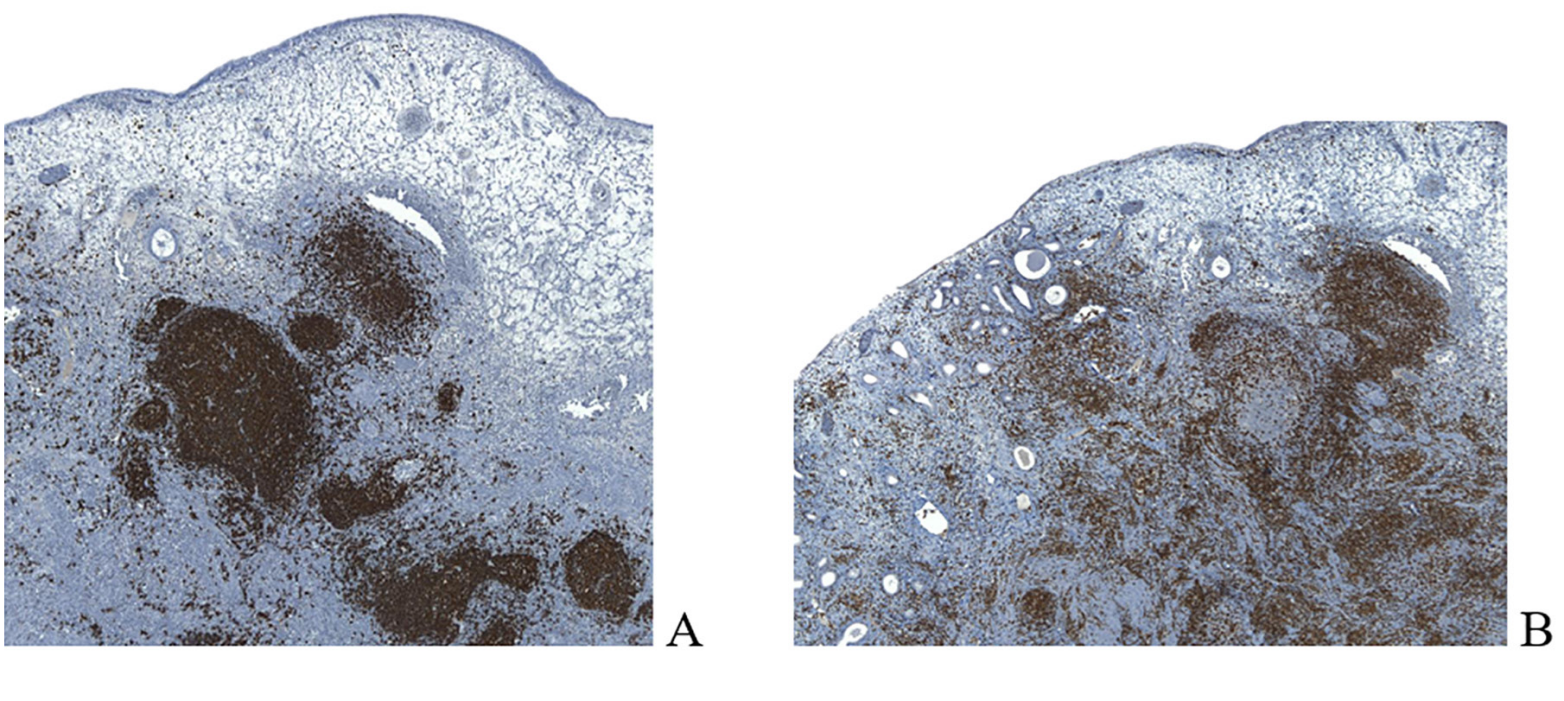
Immunohistochemistry revealed a dense population of small mature CD20 + B lymphocytes (A) with associated CD3 + T cells (B).

Immunohistochemistry demonstrated neoplastic cells positive for CD20, CD43 and BCL2, without displaying immunoreactivity for CD3, CD10, BCL6, BCL10 and CD138 (Figure 3). Ki-67 staining characterized approximately 5% of cells, revealing a low proliferation index. The patient was followed up by gynaecology and oncology teams. The gynecology follow-up included a transvaginal ultrasound examination and a hysteroscopy which showed no evidence of residual disease or recurrence. During the oncological follow-up, total body computerized tomography was performed showing no other lesions or lymphadenopathy. Therefore, the lymphoma was classified as Ann Arbor stage IE.

To the best of our knowledge, there have been only three cases of endometrial MALToma in polyps reported in the literature: two were treated with hysterectomy and one with hysteroscopic resection of the lesions (Annibali et al., 2009; De Angelis et al., 2011; Di Tucci et al., 2013). Hence, there is limited experience to recommend a gold standard therapy and follow up strategy after conservative treatment of this condition. After extensive counselling, the patient declined hysterectomy. Therefore, an individualized follow up programme was planned for this patient comprising of regular oncological and gynecological visits. After surgery, she underwent diagnostic office hysteroscopic examinations with endometrial biopsy at 6, 12, 24, 36 and 42 months, showing no endometrial abnormalities. After 42 months she is free of recurrence.

## Discussion

Primary malignant lymphomas of the genital tract are exceptionally rare neoplasms with only a few cases described in literature (Kosari et al., 2005). To this date, the largest case series of gynaecological lymphoma has been reported by Kosari et al. (2005), who found only three cases of MALToma confined to the endometrial stroma, but none discovered within a polypoid lesion.

To the best of our knowledge, this report is the fourth case of MALToma identified in the core of an endometrial polyp (Annibali et al., 2009; De Angelis et al., 2011; Di Tucci et al., 2013).

Annibali et al. (2009) first described the occurrence of MALToma inside an endometrial polyp, but the macroscopic characteristics of the lesion were not clearly reported. Later, De Angelis et al. (2011) and Di Tucci et al. (2013) described two cases of MALToma found inside both the endometrium and an endometrial polyp, also without providing a clear macroscopic description of the lesions. All other published cases of endometrial MALToma were identified in non-polypoid endometrial mucosa (Merritt et al., 2014).

It’s important to note that, when considering all four cases of MALToma (including ours) reported inside an endometrial polyp, all have been reported in postmenopausal women. As such, two patients were asymptomatic, whereas the other two presented with postmenopausal bleeding. None of the patients suffered from other conditions which can be associated with MALToma such as autoimmune or infectious diseases. (Defrancesco et al., 2017).

Our choice of conservative management warrants some consideration. In two similar cases, hysterectomy was the treatment of choice for MALToma confined to endometrial polyps, whereas Annibali et al. (2009) was the first author to report a successful outcome with conservative hysteroscopic treatment (with no recurrence of the disease after 36 months of follow up).

Since there is lack of specific recommendations for the management of endometrial MALToma, we decided to follow the National Comprehensive Cancer Network (NCCN) guidelines for non- gastric MALT lymphomas. These guidelines state that surgery might represent an appropriate initial treatment for MALToma and observation could be considered in case of negative margins after surgery (Defrancesco et al., 2017; Zelenetz et al., 2018). In this regard, we must highlight that in our patient, both the base of the polyp and the surrounding endometrium were negative for MALToma, thus, reducing the risk of recurrence. Our case is different from the other reported cases in which the presence of residual disease was confirmed by histology (i.e. positive margins) or could not be excluded (i.e. endometrial biopsies not obtained) (Annibali et al., 2009; De Angelis et al., 2011; Di Tucci et al., 2013; Merritt et al., 2014).

After opting for conservative management of a rare tumor, an optimal follow-up strategy must be planned. In our case, the patient agreed to follow-up with office diagnostic hysteroscopy and endometrial biopsy every 6 months for the first five years after surgery and, if negative, an annual follow-up. She was also encouraged to immediately alert our team in case of recurrence of postmenopausal bleeding. This follow-up strategy is generally well-received by the patients, as diagnostic hysteroscopy can be performed in an office setting with miniaturized instruments without the need for anaesthesia (Luerti et al., 2018). Nevertheless, like the other operator-dependent techniques (i.e. ultrasound), the presence of a skilled physician can maximize the effectiveness of the procedure reducing the risk of diagnostic failure (Dealberti et al., 2018).

In conclusion, MALToma is a rare condition within an apparently normal endometrial polyp. Its management must be based on the risk of residual disease and patient’s desire of preserving the uterus. When the base of the polyp as well as multiple endometrial samplings are negative for pathology, conservative management of this condition may be considered as a safe alternative to hysterectomy. If conservative management is elected, an individualized follow-up plan should be put in place to exclude disease recurrence. Overall, more data on long-term follow up of such cases is required in order to confirm the safety of the conservative management of this exceptionally rare condition.

## Consent

Informed consent was obtained from the patient to publish this case. The IRB (Institutional Review Board) was consulted for approval to publish and advised that IRB approval was not necessary for this case report.
